# The Cellular Prion Protein: A Player in Immunological Quiescence

**DOI:** 10.3389/fimmu.2015.00450

**Published:** 2015-09-02

**Authors:** Maren K. Bakkebø, Sophie Mouillet-Richard, Arild Espenes, Wilfred Goldmann, Jörg Tatzelt, Michael A. Tranulis

**Affiliations:** ^1^Department of Basic Sciences and Aquatic Medicine, Faculty of Veterinary Medicine and Biosciences, Norwegian University of Life Sciences, Oslo, Norway; ^2^UMR-S1124, INSERM, Université Paris Descartes, Sorbonne Paris Cité, Paris, France; ^3^The Roslin Institute, Royal (Dick) School of Veterinary Studies, University of Edinburgh, Edinburgh, UK; ^4^Biochemistry of Neurodegenerative Diseases, Institute of Biochemistry and Pathobiochemistry, Ruhr University Bochum, Bochum, Germany

**Keywords:** prion protein, immune privilege, immune modulation, cytoprotection, cell signaling, inflammation, neurodegeneration, Alzheimer

## Abstract

Despite intensive studies since the 1990s, the physiological role of the cellular prion protein (PrP^C^) remains elusive. Here, we present a novel concept suggesting that PrP^C^ contributes to immunological quiescence in addition to cell protection. PrP^C^ is highly expressed in diverse organs that by multiple means are particularly protected from inflammation, such as the brain, eye, placenta, pregnant uterus, and testes, while at the same time it is expressed in most cells of the lymphoreticular system. In this paradigm, PrP^C^ serves two principal roles: to modulate the inflammatory potential of immune cells and to protect vulnerable parenchymal cells against noxious insults generated through inflammation. Here, we review studies of PrP^C^ physiology in view of this concept.

## Introduction

The cellular prion protein (PrP^C^) is known for its crucial involvement, via its scrapie isoform PrP^Sc^, in the development of transmissible spongiform encephalopathies (TSEs), such as Creutzfeldt–Jakob disease in man and scrapie in sheep and goats. In these fatal diseases, the spontaneous or template-directed misfolding of PrP^C^ into abnormal PrP^Sc^ subverts PrP^C^’s normal function and causes synaptic loss and neuronal demise (1, 2). Despite extensive investigations for three decades, PrP^C^ remains a conundrum. What is the major physiological role of this protein and why does it exist under a plethora of isoforms and interact with so many partners? Why do *Prnp* knockout mice develop normally and without major phenotypic alterations, although PrP^C^ is ubiquitous and highly conserved between species? Here, based on the current knowledge of PrP^C^ function, we provide an overview of its potential contribution to a phenomenon generically known as immune privilege, providing a new angle to the question of the physiological role of PrP^C^.

During evolution of the vertebrate immune system, the potency of inflammatory responses has increased, not least during the transition from ectothermic (fish, amphibians, and reptiles) to endothermic (birds and mammals) species, dramatically increasing O_2_ consumption and reactive oxygen species (ROS) generation (3). In parallel with increasing immunological firepower, to combat intruding microorganisms and cancer cells, several anti-inflammatory and protective measures have evolved to shield “innocent bystander” cells from inflammatory damage. Interestingly, major areas of modern medical treatment are concerned with dampening inflammatory responses. In organs such as the eye, the brain, pregnant uterus, and testicles, inflammation can have devastating consequences. One fascinating development to protect such tissues with limited regenerative capacity is the evolution of immune privilege. This phenomenon is established through combinations of physical barriers and circulatory adaptations, together with organ expression of potent cell surface and soluble immunomodulatory factors (4). The principal concept presented in this review is that PrP^C^ serves an anti-inflammatory and protective role. This explains the prevalent observation that phenotypes due to loss of PrP^C^ are minor if detectable at all under physiological conditions, while clearly evident under stress and particularly under inflammation in immune-privileged organs, such as the brain.

## Immune Privilege

In the late nineteenth century, VanDooremaal and later, in the 1940s, Medawar pioneered studies that defined “immune privilege” after studying grafts that survived after being transplanted into the brain or anterior chamber of the eye (5) [reviewed in Ref. (6)]. Classically, immune-privileged sites were the central nervous system (CNS), the anterior chamber of the eye, the placenta and fetus, and testicles. Immune privilege was considered a static phenomenon, mainly resulting from anatomical and circulatory peculiarities, such as an apparent lack of lymphatic drainage. This concept was supported by the discovery of the blood–brain barrier and later the blood–testis barrier (7).

Today, our understanding of immune privilege, often referred to as immunological quiescence, is vastly extended, and may be defined as a dynamic and highly complex interplay between anatomical, physiological and immunoregulatory adaptations, which together restrict, deviate, and block inflammatory processes in the privileged tissue (8, 9). Moreover, many organs and cellular niches can obtain immune privilege, and certain elements of immune privilege have been observed in tumor growth and during chronic inflammation. Importantly, immune privilege is not a general immunosuppression, but involves tight control and often downregulation of those immune responses that potentially cause severe tissue damage. These include cytotoxic T cells, natural killer cells (NK), and pro-inflammatory cytokines.

Several cell surface and extracellular proteins play important parts in immune privilege, e.g., the immunomodulatory enzyme indolamine 2,3-dioxygenase (IDO) which causes a local depletion of L-tryptophan and thereby halts proliferation of T cells (10), the apoptosis-stimulating receptor ligand couple Fas/FasL (11), tumor necrosis factor alpha apoptosis inducing ligand (TRAIL) (12), and transforming growth factor beta (TGF-β) (13), amongst others. In addition, in many immune-privileged organs, cells, such as neurons, have low expression of major histocompatibility complex (MHC) class Ia molecules, which confer fundamental protection from cytotoxic T cells [reviewed in Ref. (4)]. They instead express non-classical MHC class Ib molecules that downregulate NK cell activity. Novel immunomodulating proteins are constantly being discovered (14, 15), and considering the importance of monitoring and fine-tuning inflammatory responses in particularly vulnerable organs, there are probably many more to come.

Another contribution to immune privilege is active recruitment of CD4^+^ CD25^+^ Foxp3^+^ regulatory T cells (TREGs), which suppress the activation of other T cells, both directly and indirectly [reviewed in Ref. (16)]. TREGs exert their immunosuppressive activities in various ways, such as cell-cycle arrest via suppressive cytokines, e.g., IL-10 and TGF-β or cell surface expression and secretion of molecules, such as Galectin-1. They can induce apoptosis in IL-2-dependent T cells via IL-2 uptake or direct cytolysis via, e.g., granzyme. Indirectly, TREGs can also exert their effects on antigen-presenting cells, impairing their co-stimulatory or antigen-presenting activity or inducing them to remain naïve.

## Posttranslational Modifications of PrP^C^

Cellular prion protein is a 210-residue glycoprotein, encoded by a single-copy gene denoted *Prnp* (17, 18). It is mainly located at the outer leaflet of the plasma membrane, attached by a glycosylphosphatidylinositol (GPI) anchor (19) and exists in many forms due to variable N-glycosylation (20) and proteolytic processing (21, 22).

**Figure 1:**
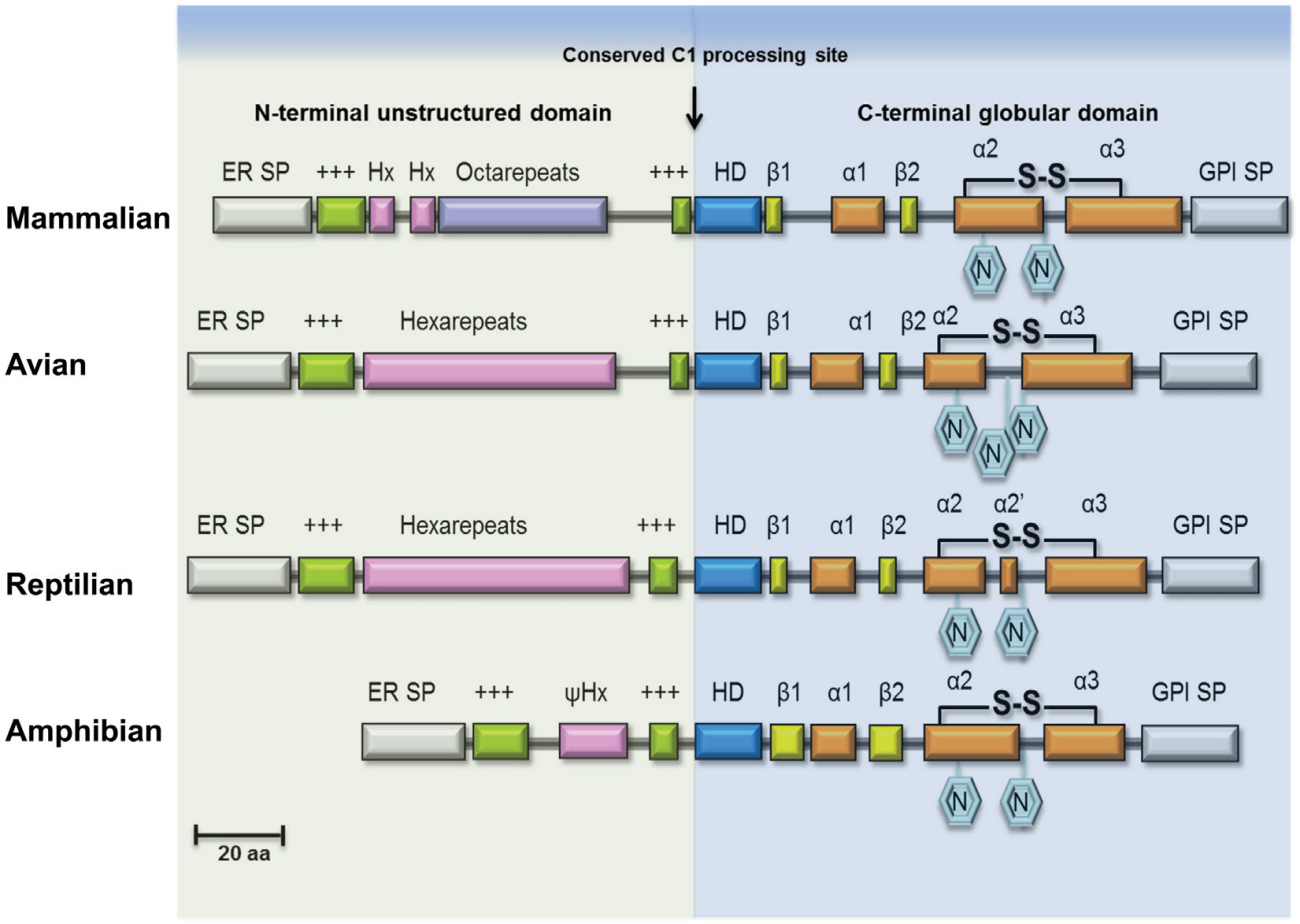
**Structural features of PrP^C^ in vertebrate lineages**. Schematic illustration of major structural features of PrP^C^ in terrestrial vertebrates. The ER transfer signal peptide (ER SP) and the glycophosphatidylinositol-anchor attachment signal peptide (GPI SP) are removed during maturation of the primary translation product, followed by N-glycosylation and cysteine bridge formation in the C-terminus. The N-terminal domain is an intrinsically disordered region (IDR) of variable length containing repeated sequence elements (pink/purple) flanked by two positively charged amino-acids motifs (green). In mammalian PrP^C^, this region contains a number of glycine-rich octapeptides or nonapeptides, preceded by two hexapeptides, while in avian and reptilian PrP^C^ it is comprised entirely of hexapeptides, and in amphibian PrP^C^ only a very short pseudo-hexapeptide (ψHx) sequence is present. A highly conserved hydrophobic, alanine-rich motif (HD) at the center is characteristic for PrP^C^. Proteolytic cleavage of PrP^C^, known as α-cleavage (arrow) occurs N-terminal to the HD-motif at the boundary between the IDR domain and the globular C-terminal domain thus generating the fragments PrP-N1 and PrP-C1 of PrP^C^ (not shown). The globular domain consists in most PrPs of three helices (α1, α2, α3) and a β-sheet formed by two short β-strands (β1, β2).

Mammalian PrP^C^ has two N-X-T sequence motifs for glycosylation (Figure 1) and most PrP^C^ isolated from tissues is indeed diglycosylated. The glycosylation sites of mammalian PrP^C^ appear invariant and they are conserved in avian, reptilian, and amphibian PrP^C^ sequences; glycosylation promotes trafficking of PrP^C^ to the plasma membrane and may increase PrP^C^ half-life. As a response to oxidative stress, mammalian PrP^C^ can be cleaved near codon 90 generating fragments PrP-N2 and PrP-C2 (22, 23). Alternatively, while passing the late-Golgi compartment, a subset of PrP^C^ is processed into two fragments PrP-N1 and PrP-C1 (Figure 1, C1 processing site) (24). The N-terminal PrP-N1 fragment is secreted, whereas the remaining C-terminal fragment PrP-C1 remains attached to the cell membrane. Whether it is localized in lipid rafts like full-length PrP^C^ remains to be shown.

PrP-C1 may also be generated on the cell membrane (23), while C-terminal shedding by ADAM10 can release full-length PrP^C^ (and PrP-C1) from the membrane (25, 26). The generation of truncated PrP-C1 is conserved in avian PrP^C^ and is likely to occur also in other vertebrates, intriguingly it is also seen in other prion family genes (*PRND, SPRN*) (27) and even in the structurally related zinc-transporter ZIP10 (*SLC39A10*) (28). Although PrP^C^ processing is in principal conserved, there is evidence that the degree of cleavage and shedding is dependent on the PrP^C^ sequence (29). Questions remain whether cleavage and PrP-C1 specifically may influence regulation and function attributed to normal prion protein and because full-length PrP^C^ and PrP-C1 are not always experimentally differentiated it is often left unresolved which of the isoforms is actually responsible for the observed activity. However, the recent finding that PrP-C1 exhibited myelinotrophic activity in the peripheral nervous system strengthens the concept that all the various PrP^C^ molecules have physiological importance (30).

## The Intrinsically Disordered N-Terminal Domain of PrP^C^

Structural studies revealed that PrP^C^ is composed of an intrinsically disordered N-terminal and a structured C-terminal domain, containing three α-helical regions and a short, two-stranded ß-sheet (31–33). Classically, the activity of a protein was linked to the ability of the polypeptide chain to adopt a stable secondary/tertiary structure. This concept was extended when it became evident that intrinsically disordered region (IDRs) and proteins can participate in a broad range of defined physiological activities and play a major role in several protein classes, including transcription factors, scaffold proteins, and signaling molecules (34–36). This ability of IDRs to interact with many different substrates may explain the observation that PrP^C^ can flexibly engage in a variety of supramolecular complexes (see below).

Considering the evolution of PrP^C^, it is interesting to note that the three-dimensional structure of the globular C-terminal domain of human (121–230), chicken (121–225), turtle (121–225), and xenopus (90–222) PrP^C^ show extensive similarities, indicating a conserved activity (37). By contrast, the N-terminal IDR of PrP^C^ shows high diversity between vertebrate classes. This is in line with the observation that IDRs often evolve more rapidly than well-structured protein domains (38). Many IDRs show sequence conservation, sometimes involving particular amino-acid residues, such as leucine (L), proline (P), tyrosine (Y), and tryptophan (W) (39). The IDR of PrP^C^ harbors many of these characteristics. Specifically, mammalian PrP^C^s contain glycine-rich octarepeats with conserved W, P, and histidine (H) residues in this region (18, 40–46), while a hexarepeat region with conserved P, H, and Y is found in avian (47–49) and reptile PrP^C^s (50). Strikingly, amphibian PrP^C^ is devoid of any repeats and H residues (51). Interestingly, IDRs are prevalent in virus proteins, allowing many interacting partners. Correspondingly, many proteins involved in innate immunity also carry IDRs, which may reflect the evolutionary “arms” race between invading pathogens and the host immune system. The evolutionary modifications that can be observed in the N-terminal IDR of PrP^C^ among terrestrial vertebrates may indeed be a relic of these evolving immune functions. The most apparent evolutionary change that has occurred first in some reptilian species and then has become the norm in avian and mammalian PrP^C^ are precisely spaced H residues which allow binding of divalent metal-ions, such as Cu^2+^ (52) and Zn^2+^ (53). Cu^2+^ binding will not only confer structural order to the N-terminus (54) but also by operating as a sensitive regulator of the structural state of PrP^C^’s IDR it may govern protein interactions and proteolytic processing (PrP-N1/PrP-C1).

Studies, comparing wild-type PrP^C^ with mutated PrPs lacking the repeat region have shown that the octarepeat region is crucial for PrP^C^’s neuroprotective activity. For instance against Bax induced cell death (55) or toxicity caused by ectopic expression of the PrP-like protein Doppel (Dpl) (56, 57), excitotoxic stress and PrP^Sc^ toxicity (58). Interestingly, Drisaldi and co-workers demonstrated that the neuroprotective function of the repeat region is dependent on four histidine residues (57). Furthermore, the repeat region is also necessary for the PrP-mediated neuroprotection observed in models of brain ischemia (59). Similarly, the ability of PrP^C^ to transmit neurotoxic signaling of amyloid beta (Aβ) and other neurotoxic β-sheet-rich-conformers is greatly diminished in PrP^C^ mutants devoid of this domain (60). Further studies of the mechanisms underlying the activities of the IDR of PrP^C^ and its evolution, particularly in neuro-immune crosstalk appears to be an important area for future research.

Functionally, PrP^C^ can by virtue of its GPI anchor move between membrane subdomains (61, 62), and interact with many partners at the cell surface. These partners may include other GPI-anchored molecules like the proteoglycan Glypican-1 (63), transmembrane proteins like the neural cell-adhesion molecule, NCAM (64), the low-density-related protein LRP1 (65), the amyloid precursor protein APP (64, 66), lipid raft constituents such as caveolin (67), or src kinases (68, 69). The formation of these complexes may occur following the interaction of PrP^C^ with extracellular matrix components, e.g., vitronectin (70) or laminin (71) or soluble ligands such as the extracellular chaperone stress-induced phosphoprotein 1 (STI1) (72). Moreover, in lymphoid cells PrP^C^ has been shown to be recruited into microdomains of the membrane, the so-called immunological synapses harboring T-cell receptor components (73–76). As discussed above, many ligands appear to interact with the N-terminal IDR of PrP^C^ [reviewed in Ref. (77)].

Of major pathophysiological relevance is the ability of the N-terminal PrP fragment (PrP-N1) to bind to and to mediate toxic effects of Aβ oligomers (78). PrP^C^ may further engage into homophilic interactions or bind the two other members of the prion protein family Doppel and Shadoo (58, 68). It could be speculated that shed PrP^C^ or PrP-N1 can act at a distance via extracellular fluids. Indeed it has been demonstrated that soluble PrP-N1 fragments can prevent Aβ-induced toxicity and have a neuroprotective activity (79–84).

## PrP^C^ Pattern of Expression

**Figure 2:**
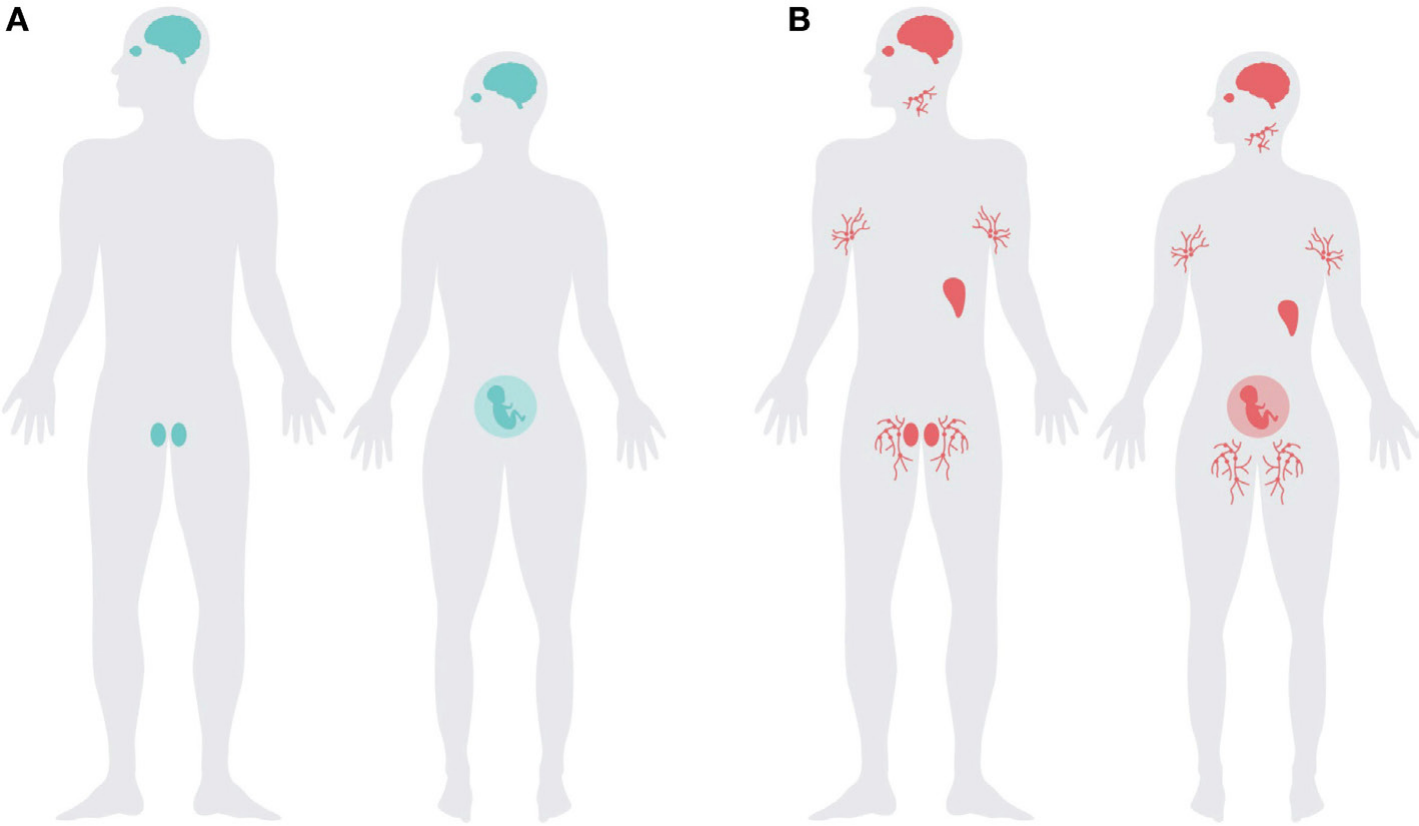
**The expression pattern of *Prnp* overlaps with immune-privileged organs and extends into the lymphoreticular system**. Schematic representation of **(A)** major immune-privileged organs; highlighting the brain, testes, pregnant uterus, and eye, and **(B)** the organs with highest levels of PrP^C^ overlap with the immune-privileged organs with the addition of lymphoreticular tissues, illustrated with lymph nodes and the spleen.

Although PrP^C^ is ubiquitously expressed, its main expression overlaps strikingly with the distribution of immunologically quiescent sites (Figure 2). PrP^C^ is abundantly present in the central and peripheral nervous system (17, 18, 85), glial cells of the CNS (86, 87), and in the testes, eye, placenta, and uterus (88, 89). PrP^C^ is also present in the neurovascular unit, including endothelial cells (90, 91), it may thus be one of the protagonists modulating blood–brain barrier functions (92).

Cellular prion protein is also found in microglial cells (93). Whether its expression is associated with the inflammatory M1 producing TNF-α, IL-1β, and IL-6, or the immunosuppressive M2 (producing IL-10 and TGF-β) phenotype of microglia has not been clarified. Notwithstanding, the observation that *Prnp* knockout mice produce less of the anti-inflammatory cytokine IL-10 in response to LPS-induced chronic inflammation would suggest a positive role for PrP^C^ in M2 microglia (94).

Cellular prion protein is abundantly expressed in neuronal and non-neuronal stem cells, including hematopoietic stem cells (HSCs) (95) and it contributes to stem-cell renewal, reviewed by Lopes and Santos (96) and Martin-Lannerée et al. (97). HSCs have been shown to co-localize with TREGs, suggesting that TREGs provide a “shield” conferring relative immune privilege to the HSCs (98). Interestingly, PrP^C^ is found at high levels in both immature HSCs (95) and TREGs (99), and probably contributes to the interplay between HSCs and TREG cells in this niche.

Differentiation of HSCs along the lymphoid (100–102) or monocytic (103) lineages maintains the expression of surface PrP^C^, while during the granulocyte maturation PrP^C^ is downregulated (104). Within lymphoid cells, B cells express lower levels of PrP^C^ compared to T cells and NK cells (105), which could be linked to the observation that B cells are not repressed in immune-privileged sites. Regulatory CD4^+^ CD25^+^ T cells expressed 4.5 fold higher levels of *Prnp* mRNA and showed a 10-fold higher intensity of surface PrP^C^ than CD4^+^ CD25^−^ T cells (99). However, an attempt to identify the role of PrP^C^ in TREGs using *Prnp* knockout mice was unsuccessful since no loss-of-function phenotypes could be recognized in Tregs without PrP^C^ expression (99). In most immune cells, PrP^C^ is dynamically expressed and generally naïve immune cells contain less PrP^C^ than mature or stimulated immune cells, with a few exceptions (105, 106). Even neutrophils strongly upregulate PrP^C^ levels after treatment with immunosuppressive TGF-β or dexamethasone (107). On some immune cells, such as mast cells, *Prnp* is highly and constitutively expressed and PrP^C^ is rapidly released from the cell surface when these cells are activated (108).

Following from the concept presented here, the principal role of PrP^C^ in immune-privileged organs would be to protect against inflammatory damage. The dynamically regulated levels of PrP^C^ observed in cells of the immune system, particularly the high expression in immunosuppressive TREGs, indicate that PrP^C^ is important in immunological homeostasis.

## Inflammation Reveals Cytoprotective and Immunomodulatory Roles of PrP^C^

*Prnp* knockout mice develop normally, with normal life expectancy (109, 110) and exhibit complete resistance toward prion infection. Despite a relative lack of robust and reproducible phenotypes under physiological conditions, a wide variety of roles for PrP^C^ have been suggested, such as in maintenance of axonal myelin (30, 111), modulating circadian rhythms (112), and neuronal excitability (113). For a comprehensive review of suggested physiological roles of PrP^C^, see Ref. (114). In addition to murine models, *Prnp* knockout cattle have been produced (115). After extensive analysis, under physiological conditions, only minor phenotypes were observed. Similar findings have been reported from a recently discovered line of Norwegian dairy goats, carrying a nonsense mutation that renders these animals devoid of PrP^C^ (116, 117).

Interestingly, experiments with *Prnp* knockout mice involving a diverse set of inflammatory processes (Table 1), such as experimental brain ischemia, brain trauma, experimental autoimmune encephalomyelitis (EAE), experimental colitis, and, intracerebral infection with encephalomyocarditis virus variant B (EMCV-B), have revealed that in the absence of PrP^C^, inflammatory damage is greatly exacerbated; reviewed by Onodera et al. (118).

**Table 1:** **Loss of PrP^C^ aggravates immunopathology in a variety of experimental settings**.

	Tissue damage^a^		
	
Insult	*Prnp* wild type	*Prnp* knockout	Reference
Brain ischemia	**++**	**++++**	(59, 119–122)
Brain trauma	**++**	**++++**	(123, 124)
Experimental autoimmune encephalomyelitis (EAE)	**++**	**++++**	(125, 126)
Experimental colitis	**++**	**++++**	(127)
Encephalomyocarditis Virus variant B	**++**	**++++**	(128)

*^a^Refers to onset, duration, and severity of inflammation and magnitude of tissue damage*.

Experimental autoimmune encephalomyelitis in mice, a chronic demyelinating disease of the CNS and a model of multiple sclerosis in humans, is often induced by immunization with myelin oligodendrocyte glycoprotein (MOG). Autoantigen-specific T cells of both the Th1 and Th17 phenotypes cross the BBB and are the primary immune cells recruited to the CNS where they activate microglia and attract blood monocytes and other inflammatory cells (129). Induction of EAE by MOG injection in *Prnp* knockout mice resulted in earlier onset, prolonged and more severe neuroinflammation than in normal mice (125). The *Prnp* knockout mice had persisting T-cell and monocytic/microglial infiltrates in the CNS, accompanied by demyelination and axonal drop-out in spinal cord white matter. It was concluded that PrP^C^ modulates T-cell-mediated neuroinflammation, with a suppressive effect on MOG-induced peripheral T-cell responses and the authors discussed whether the larger pathological lesions in mice lacking PrP^C^ also could be caused by increased cellular susceptibility to oxidative stress.

Gourdain and colleagues (126) conducted experiments with reciprocal bone marrow chimeras with lack of PrP^C^ expression in lymphoid cells or the CNS, but did not observe earlier disease onset nor increased leukocyte infiltration in the CNS in animals with *Prnp* knockout lymphocytes. However, they observed significantly higher pathology scores in mice lacking PrP^C^ expression in the brain, and concluded that PrP^C^ primarily confers neuroprotection against neuroinflammatory insult. In a different attempt to discriminate neuroprotection by PrP^C^ from immunoregulatory roles, Hu and co-workers (130) used pharmacologically selective silencing of PrP^C^ in lymphocytes in models of nervous system autoimmune disease. They were able to show that depletion of PrP^C^ in lymphocytes directly affected T-cell activation, survival, and differentiation. In the absence of PrP^C^ expression in lymphocytes, the severity of EAE was considerably increased. They concluded that lack of PrP^C^ in lymphocytes resulted in pro-inflammatory activities and that autoimmune brain pathologies could develop despite protective PrP^C^ expression in neuronal cells. Thus, under these experimental conditions, the role for PrP^C^ as a regulator of immunological homeostasis apparently dominated the cytoprotective role of the protein in the CNS. In Gourdain et al. (126), the authors state that their data do not exclude an important role for PrP^C^, particularly in early lymphoid responses. They further discuss several possible explanations for the conflicting results in their study and the study by Hu et al. (130). The models used are complicated and obviously differ in many aspects, such as mouse strain, encephalitogenic antigens, T-cell assay protocols and differences in methodology for gene silencing, all factors that may have contributed to the discrepancies.

In a different model with induction of focal brain ischemia, *Prnp* knockout mice experience more severe tissue damage after focal brain ischemia than wild-type mice (59, 119–121). Spudich and colleagues observed 200% larger infarct volume in *Prnp* knockout mice. In addition, an induction of ERK1/2, STAT1, JNK1/2, and Caspase-3 activity in the *Prnp* knockouts suggests that these signaling molecules are involved in *Prnp* knockout-related neuronal cell death (120). Significantly increased infarction volumes were also observed in *Prnp* knockout mice, in studies of permanent and transient focal cerebral ischemia (121). Moreover, they observed reduced levels of phosphorylated Akt and enhanced neuronal caspase-3 activation in *Prnp* knockout mice. In a rat stroke model, Shyu and colleagues revealed a time-dependent increase in PrP^C^ levels in infarcted tissue to reach a peak 3 days post infarction and that overexpression of PrP^C^ reduced ischemic injury (122). Mitsios and colleagues detected increased levels of PrP^C^ in plasma from human stroke patients compared with healthy controls (131). Moreover, they found upregulation of PrP^C^ in gray matter peri-infarcted tissue and in infarcted tissue, in 6 out of 10 patients. In a study of traumatic brain injury, *Prnp* knockout mice had a larger lesion volume and a breakdown of the blood–brain barrier 1 month after injury compared to wild-type mice (123). Interestingly, it has been observed that *Prnp* mRNA is one of two most upregulated mRNAs after induced traumatic brain injury in mice (124), supporting a protective role for PrP^C^. From the studies on ischemic and traumatic brain injury, the role of inflammation in the development of lesions remains to be clarified, since no inflammation-specific parameters were measured, nor infiltration of cells along the borders of the necrotic tissue were observed. Cell death also activates inflammation and further studies should include the role of cytokines and immune cells, such as locally activated or blood-derived macrophages/microglia, when evaluating how PrP^C^ influences damage control (94).

Intracranial infection with EMCV-B resulted in similar viral titers in wild-type and *Prnp* knockout mice, however, mice lacking PrP^C^ showed higher numbers of apoptotic neurons, while wild-type mice had more activation of microglial cells as well as more severe infiltration of immune cells in the hippocampal area (128), suggesting that PrP^C^ affected the inflammatory response, while also serving a protective, anti-apoptotic role during the infection.

In a mouse model of inflammatory bowel disease, mice lacking PrP^C^ developed a more severe colitis with markedly elevated levels of pro-inflammatory cytokines and pro-apoptotic regulatory proteins (127). Moreover, it was shown that overexpression of PrP^C^ protected against induction of colitis. Interestingly, lack of PrP^C^ has been shown to skew T-cell development in favor of pro-inflammatory Th1 and Th17 phenotypes (125, 130).

Taken together, these observations made under different experimental modalities demonstrate that PrP^C^ both mediates cytoprotective signaling under inflammatory stress and has the capacity to attenuate the inflammation itself.

In summary, PrP^C^ is highly expressed in immune-privileged organs and it serves a protective role evident most clearly under inflammatory stress and/or tissue damage. Moreover, data show that PrP^C^ is more than a passive protector, but also dampens the inflammation itself, by modulating the activity of immune cells in an anti-inflammatory direction. The latter role of PrP^C^ fits well into the concept of immune privilege and immune modulation. The molecular details and signaling pathways by which PrP^C^ modulates inflammation are not yet clarified and stand out as a challenging area of future research. Below, we highlight some of the current data on signaling mediated via or influenced by PrP^C^ presence on the cell surface.

## PrP^C^ in Cytoprotective and Immunoregulatory Signaling

**Figure 3:**
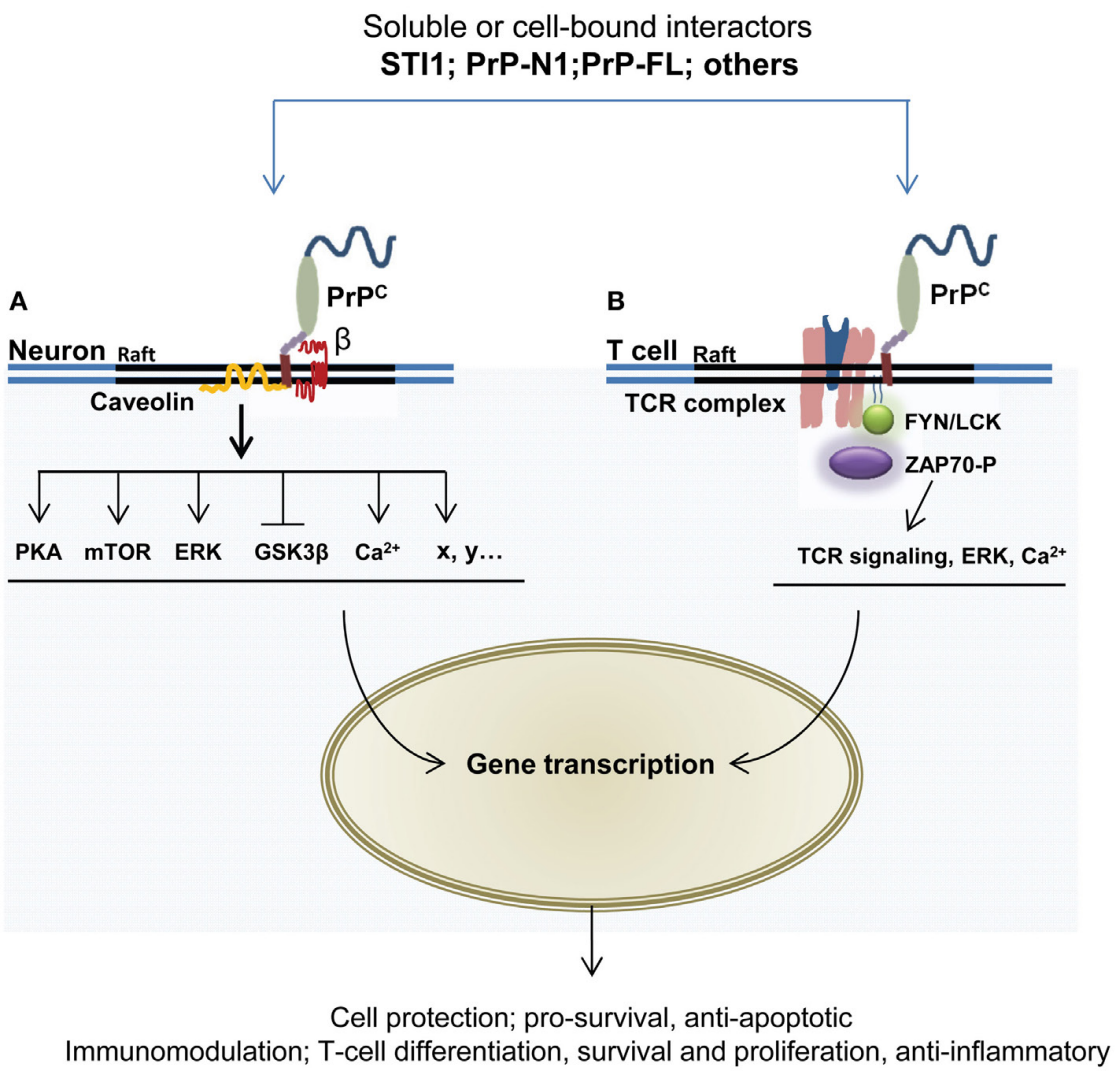
**GPI-anchored PrP^C^ has the capacity to interact with multiple partners and trigger cell signaling events leading to neuroprotection and immunomodulation**. The formation of PrP^C^-containing multimolecular complexes and subsequent mobilization of protective or immunomodulatory signals may be induced following the binding of soluble ligands, such as STI1, PrP^C^ itself, or its N-terminal domain (PrP-N1). **(A)** In neurons, several targets of PrP^C^ signaling relay its neuroprotective action, including protein kinase A, mTOR, the MAP kinases ERK1/2, and GSK3b. This action may also involve intracellular calcium mobilization. Some of these signaling cascades require the association of PrP^C^ with the membrane protein caveolin. The participation of transmembrane partner(s) of PrP^C^ to the signaling complexes is represented by «β». **(B)** In T lymphocytes, PrP^C^ is enriched at the immunological synapse, where it can interact with components of the T cell receptor (TCR), such as the Fyn tyrosine kinase and the zeta chain-associated protein kinase 70 (ZAP-70). These interactions may further promote the activation of downstream effectors, including NFkB, JNK, ERK, as well as elevation of intracellular calcium concentration.

At a molecular level, the cytoprotective activity of PrP^C^ may depend on its capacity to engage into multimolecular complexes at the cell surface and mobilize signal transduction cascades. For a review of these signaling events in neuronal cells, see Ref. (132). With respect to the concept presented here, the molecular cascades underlying the potential contribution of PrP^C^ to immune quiescence remain to be dissected. However, many of these probably overlap with cytoprotective signaling, which is better characterized. We will therefore elaborate somewhat on PrP^C^ partners and effectors potentially contributing to its cytoprotective activity (Figure 3). The extracellular co-chaperone STI1, identified as a PrP^C^ partner in 1997 through a complementary hydropathy approach (133), is a well-established inducer of PrP^C^-dependent signals. The STI1–PrP^C^ interaction has been shown to protect retinal (72, 134) and hippocampal neurons (135) against chemically induced apoptosis, in both cases via cAMP-dependent protein kinase A (PKA). The neuroprotective action of this partnership is also supported by the recruitment of mTOR (136) as well as the inactivation of the GSK3β kinase (69), whose overactivity is detrimental to neurons (137). Noteworthy, STI1 can be secreted from astrocytes (138), a cell type highly contributing to immune quiescence in the CNS (92). STI1 may act in a cell-autonomous manner to favor astrocytic differentiation upon binding to PrP^C^ (139). Whether the STI1–PrP^C^ interaction also instigates a dialog between astrocytes and neurons deserves to be considered. Interestingly, astrocytes release STI1 in response to oxygen–glucose deprivation (140), and thereby induce neuroprotective signals through PrP^C^. In line with this, Lee and colleagues found that STI1 is induced in the ischemic brain and contributes to recovery via PrP^C^ (141). The same study showed that the upregulation of STI1 promotes the recruitment of bone marrow-derived cells to the ischemic brain and thereby helps reducing brain injury. Although the full pathway of signaling events imparted by the STI1–PrP^C^ duo in this context remains to be elucidated, a beneficial contribution of the downregulation of matrix-metallopeptidase 9 (MMP-9) transcripts and activity fostered by PrP^C^ (142) should be proposed, since MMP-9 knockout mice are less vulnerable to ischemia than their wild-type counterparts (143), possibly because activation of MMPs during brain injury leads to increased permeability of the glia limitans (144), separating the perivascular space from the neural tissue proper and thereby opens for a higher flux of cells and solutes into the neuropil.

In addition to the mature brain, the cytoprotective action of the PrP^C^–STI1 interaction may operate during embryonic development since the two proteins are expressed from early stages (140, 145). Beraldo and colleagues reported that maternally derived STI1 can be found in blastocysts. Deletion of the STI1 gene in mice leads to embryonic lethality around E9–E10.5 due to placental disruption and lack of cellular viability (140). The molecular details causing embryonal lethality in the STI1 knockout mice have not been elucidated. This drastic phenotype implies that STI1 signaling in early embryos is relayed by several receptors beyond PrP^C^. These include the activin receptor-like kinase-2 (ALK2) (146), whose deficiency leads to developmental arrest at the gastrulation stage (147). Another possibility to be considered is the binding of STI1 to the PrP^C^ homolog Shadoo, which is abundantly expressed in extra-embryonic annexes, and which may have overlapping functions with PrP^C^ during early development (148). Notwithstanding, because PrP^C^ is abundant in extra-embryonic annexes (148), STI1–PrP^C^ protective signaling is likely to also occur in placenta, another important site of immune privilege. With respect to embryonic development, it is worth noting that the binding of STI1 to PrP^C^ enhances the self-renewal of neural progenitor cells (149). On the other side, however, STI1–PrP^C^ signaling appears to contribute to tumor growth (150) (see below).

Regarding cells of the immunological lineage, it has long been known that PrP^C^ is present on the surface of lymphocytes and that it is rapidly upregulated upon activation of these cells (100–102). Mattei and co-workers demonstrated that PrP^C^ was part of a signaling complex involved in T-cell activation (73). Furthermore, through immunoprecipitation they observed an association between PrP^C^ and the signaling tyrosine kinase Fyn, and, in activated T cells, PrP^C^ was co-immunoprecipitated with zeta chain-associated protein kinase 70 (ZAP-70), a protein of central importance in T-cell receptor signaling. Upon silencing of PrP^C^ in lymphocytes by *Prnp* siRNA, an increase in ZAP-70 activation was seen with a corresponding rise in CD3/CD28 which was stimulated by transcription factor NFAT/AP-1, known for its function in T-cell signaling and differentiation (130). In Jurkat lymphocytes, a co-localization of PrP^C^ and T-cell co-receptor CD3ϵ and the lipid-raft ganglioside GM1 was observed (74, 76). Further compelling evidence of PrP^C^ operating in immunological synapses, such as in antigen-driven interactions between T cells and DCs, have been reported (75). Interestingly, absence of PrP^C^ in T cells and DCs had different consequences for T-cell proliferation; T cells devoid of PrP^C^ exhibited a normal allogenic antigen response, while DCs without PrP^C^ significantly reduced proliferation in interacting T cells, suggesting that PrP^C^ might serve different signaling roles in the two cell types. From experiments using PrP^C^ antibodies, the authors concluded that PrP^C^ is a negative regulator of T-cell receptor signaling and that PrP^C^ modulates neuroinflammation.

## PrP^C^ and Cancer

While immune privilege represents a physiological safeguard mechanism, it is also known to be hijacked by cancer cells to evade antitumor immunity (151). Over a decade ago, PrP^C^ was found to be overexpressed in a breast cancer cell line that was resistant to TNFα-induced cell death (152). A correlation between PrP^C^ expression and resistance to cytotoxic agents has now been described in various types of tumors, including breast cancer, gastric cancer, and glioblastoma [reviewed in Ref. (153)]. While the molecular mechanisms underlying the contribution of PrP^C^ to tumor resistance are poorly understood, the disruption of the STI1 binding to PrP^C^ was recently shown to impair glioblastoma growth (150), suggesting that cancer cells may usurp the cytoprotective activity of PrP^C^. A question that deserves further investigation is whether the presence of PrP^C^ at the surface of cancer cells endows them with properties that enable them to evade the immune response.

## Future Prospects

Although the concept presented here allows many pieces of the PrP^C^ puzzle to fall into place, by providing principal physiological roles of PrP^C^ in all tissues, several important questions remain to be answered. For instance, does surface-bound PrP^C^ on patrolling immune cells interact with PrP^C^ or a PrP^C^-controlled protein complex in tissues and cells, like in the blood–brain barrier tight junctions of the endothelial cells (90) and thereby sense the entrance into an immune-privileged, PrP^C^-enriched zone; thus contributing to “do no harm” signaling? To what extent does PrP^C^ play part in the maintenance of stem-cell niches, in the proliferation and differentiation of cell lineages derived from the bone marrow and in modulating the development of lymphoid organs? Furthermore, the concept presented here calls for careful scrutiny of the role of PrP^C^ in chronic inflammatory conditions, such as inflammatory bowel disease and various pathologies eliciting inflammation in the brain or other immune-privileged organs.

Besides, in all these questions, we have to examine which of the many PrP^C^ protein/peptide forms are the actual executors of these physiological roles. From the genomic perspective, it would be of great help to understand the gene control of *Prnp* and whether other immunomodulators, such as TRAIL, Fas/FasL, and IDO, are part of the same expression network controlled by similar transcription factors; and whether there is genetic variation resulting in altered immune-privilege. Our proposed link between PrP^C^ and immunological quiescence opens an exciting new avenue for the study of this protein beyond the chronic diseases of the CNS into the domain of the immune system, the reproductive system, and in sensatory organs. It will encourage the study of PrP^C^ in several inflammatory conditions and cancer.

## Conflict of Interest Statement

The authors declare that the research was conducted in the absence of any commercial or financial relationships that could be construed as a potential conflict of interest.

## Funding

MB, AE, and MT were supported by funds from the Research Council of Norway and the Norwegian University of Life Sciences. SM-R is supported by funds from INSERM. JT is supported by grants from the Deutsche Forschungsgemeinschaft and the Max Planck Society. WG is supported by a BBSRC UK Strategic Programme Grant to The Roslin Institute.
